# Moderate acute alcohol intoxication increases visual motion repulsion

**DOI:** 10.1038/s41598-018-19932-8

**Published:** 2018-01-25

**Authors:** Zhengchun Wang, Huan Wang, Tzvetomir Tzvetanov, Yifeng Zhou

**Affiliations:** 1 Department of Physiology and Pharmacology, Medical School of Ningbo University, Ningbo, Zhejiang 315211 P.R. China; 2 Hefei National Laboratory for Physical Sciences at Microscale and School of Life Science, University of Science and Technology of China, Hefei, Anhui 230027 P.R. China; 3 School of Computer and Information, Hefei University of Technology, Hefei, Anhui 230009 P.R. China; 4 State Key Laboratory of Brain and Cognitive Science, Institute of Biophysics, Chinese Academy of Science, Beijing, 100101 P.R. China

**Keywords:** Perception, Visual system

## Abstract

Among the serious consequences of alcohol abuse is the reduced ability to process visual information. Diminished vision from excessive consumption of alcohol has been implicated in industrial, home, and automobile accidents. Alcohol is also generally recognized as an inhibitor in the brain by potentiating GABA-ergic transmission. In this study, we focused on visual motion processing and explored whether moderate alcohol intoxication induced changes in inhibitory mediated motion repulsion in a center-surround configuration. We conducted a double-blind, placebo-controlled, within-subjects study on the effect of alcohol on visual motion repulsion. Each subject underwent three experimental conditions (no alcohol, placebo and moderate alcohol) on separate days. The order of the placebo and moderate alcohol conditions was counterbalanced. The results showed that the effects of the surround context on the perception of the center motion direction were similar in both the sober (no alcohol) and placebo conditions. However, contextual modulations were significantly stronger during intoxication compared to both the sober and placebo conditions. These results demonstrate that moderate alcohol consumption is associated with altered neural function in visual cortical areas and that motion repulsion deficits might reflect the inhibitory effects of alcohol on the central nervous system.

## Introduction

Acute alcohol consumption disturbs cognitive, attentional, motor, and perceptual functions in a dose-dependent manner. Negative effects occur with a small to moderate blood alcohol concentration (BAC) of approximately 0.4–0.5 mg/ml^1–3^. A moderate BAC affects sensory functioning. For example, it has been reported that the social and drinking profiles of road crash casualties with BACs greater than 0.5 mg/ml differ from those of other casualties. Diminished vision from excessive consumption of alcohol has been implicated in industrial, home, and automobile accidents^4^. Additionally, deficits in visual perceptual functions have been widely identified in alcohol intoxication. For example, contrast sensitivity is reduced with a BAC of 0.43 mg/ml^5^. This deficit is more pronounced with higher dosages of alcohol and depends on the presentation time of the test stimuli^6,7^. Acute alcohol intake also significantly changes the processing of contrast sensitivity under certain frequencies compared with control conditions^8^.

Similar to visual perceptual deficits at the behavioral level, visually evoked potential (VEP) amplitudes are reduced during the processing of contrast in alcohol intoxication^9^. Compared to the placebo condition, alcohol intoxication was shown to strongly attenuate early visual activity occipito-temporally in an anatomically constrained MEG study^10^. Alcohol is also associated with deficits in inhibitory systems through potentiation of GABA-ergic transmission in many cortical areas^11^. Low to moderate concentrations of alcohol have been shown to enhance GABA-ergic inhibition, specifically by enhancing GABA-A receptor function^12^. In fact, GABA agonists and reuptake inhibitors have been shown to enhance the effects of alcohol, whereas GABA antagonists have been shown to reduce the effects of alcohol^13^. However, the functional relevance of impaired inhibitory processing on visual perceptual dysfunction in acute alcohol ingestion remains elusive.

**Figure 1:**
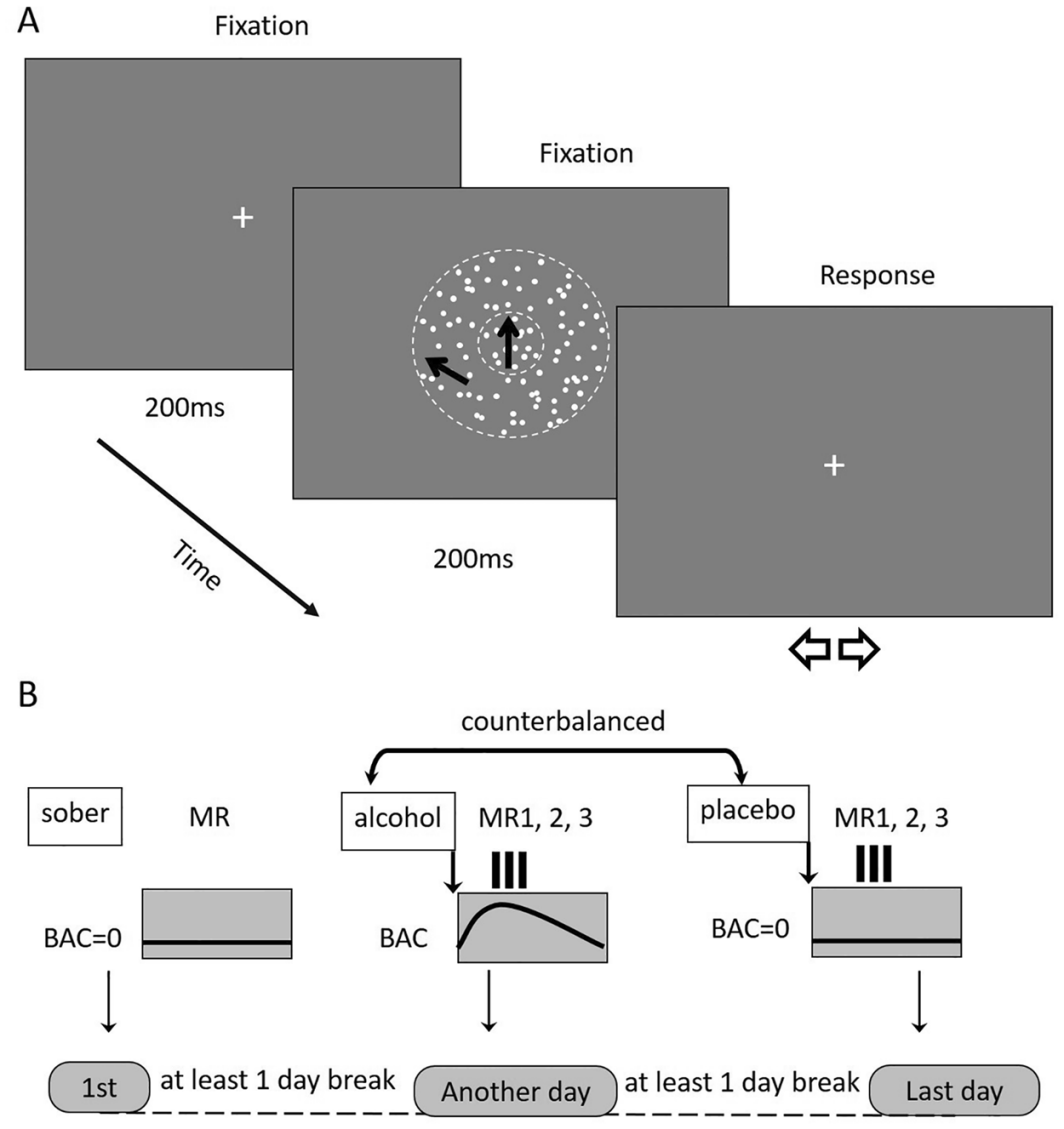
Direction discrimination task and experimental design. (**A**) A trial example. Subjects pressed a predefined key to start the trial. Center-surround moving dot patches were presented for 200 ms after a 200 ms fixation. Observers were required to report whether the motion direction of the center dots was clockwise (CW) or counter clockwise (CCW) from the internal vertical standard by pressing two predefined keys. The blank window was sustained until the subjects responded. The black arrows show the direction of motion in the corresponding part of the stimulus. In this example, the central target contained 100% coherent motion in the vertical upward direction and the surround annulus contained 100% coherent motion at the −60° (CCW) diagonal direction. The direction difference between the center target and surround was 60°. (**B**) Experimental design. Sober (no alcohol), placebo and alcohol conditions were conducted on separate days. Each subject began with a BAC measure, and then, motion repulsion measurements were conducted three times in both the placebo and alcohol conditions.

One compelling phenomenon that inhibits visual information processing is motion repulsion (MR)^14–16^. MR is characterized by the concept of illusory motion perception, where human performance in a motion discrimination task shows a perceptual misjudgment of the target direction due to irrelevant motion in the spatial surround of the target stimulus (Fig. 1). The paradigm is known to give rise to motion repulsion, reflecting a systematic overestimation of the angular difference between two motion directions^14,17^. In particular, subjects misperceive the physical direction of the target motion when the task is irrelevant and the target moves at directions that are approximately 30–60° away from the target direction. Motion repulsion is commonly interpreted through lateral inhibitory interactions between motion sensitive neurons^17^. Motion repulsion falls into the same category as the surround suppression effect, which is believed to be important for visually detecting edges and determining the salience of image features during camouflage^18^.

Therefore, we hypothesized that inhibition-based motion repulsion is altered by moderate concentrations of alcohol. Potentiation of inhibitory mechanisms should increase the magnitude of motion repulsion. We employed a double-blind, placebo-controlled, within-subjects study of the effect of alcohol on motion repulsion. Each subject was exposed to three experimental conditions (no alcohol, placebo and moderate alcohol) on separate days. The dose of moderate alcohol was approximately 0.6 mg/ml. The order of the conditions was counterbalanced, and we compared the magnitudes of the motion repulsion of participants for the three conditions.

## Results

### Breath alcohol analyses

**Figure 2:**
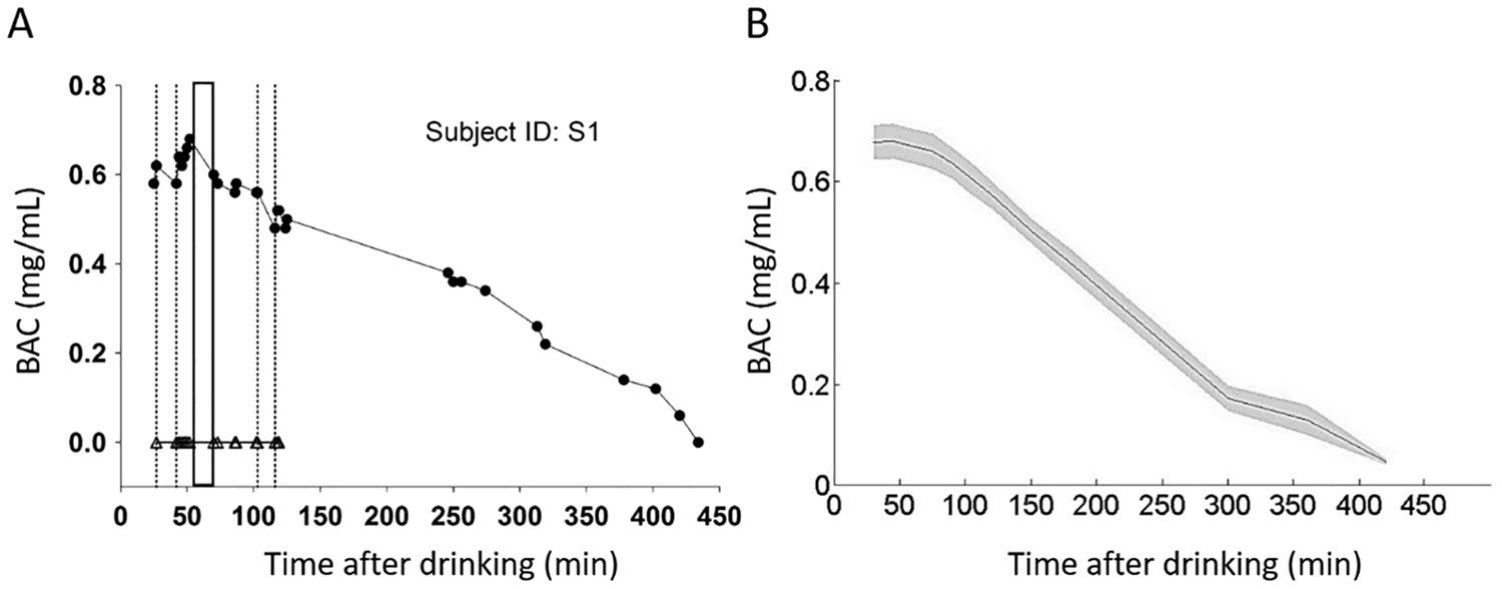
Profiles of alcohol metabolism for an example subject and population. (**A**) Complete set of BAC data for subject S1 in the alcohol condition and placebo condition. BAC recording began approximately 30 min after alcohol/placebo beverage administration; the subject completed the direction discrimination task 3 times after alcohol/placebo beverage intake (dotted and solid rectangles delineate the time window of measure). The second measure (solid square), closer to the peak BAC, was used for analysis of the alcohol condition. Triangles represent BACs for the placebo condition. (**B**) BAC values across all subjects over time. The solid square indicates the mean measurement times of the included motion repulsion data in the alcohol condition. The solid curve and grey shadow indicate the mean ± SE.

BAC levels were measured continuously after alcohol administration to determine the alcohol metabolic process of each subject and were recorded directly before and after task performance. On average, the time to complete the motion direction discrimination task was approximately ten minutes, allowing subjects enough time to repeat the same task three times after alcohol intake. The alcohol metabolic curve of each participant was fitted with a Widmark function. Thus, the MR results shown below were obtained at approximately the peak BAC value for each subject. In the sober (no alcohol) and placebo states, all participants started and completed task performance with a BAC of 0 mg/ml. When the task was initiated in the intoxicated state, the participants had a mean BAC of 0.66 ± 0.03 mg/ml. Immediately after the measurement of interest, the mean BAC was 0.64 ± 0.03 mg/ml. A t-test revealed that the intoxication difference (pre versus post task performance) was not significant (t [27] = 1.40, p = 0.173) See Fig. 2 for an example of an individual BAC (A) as well as for the population mean BAC curve (B). ANOVA showed that there were no significant differences in the BAC values between the three measures (mean ± SD for first 0.66 ± 0.18, second 0.64 ± 0.17 and third 0.60 ± 0.13, p = 0.20).

### Increased motion repulsion after alcohol administration

**Figure 3:**
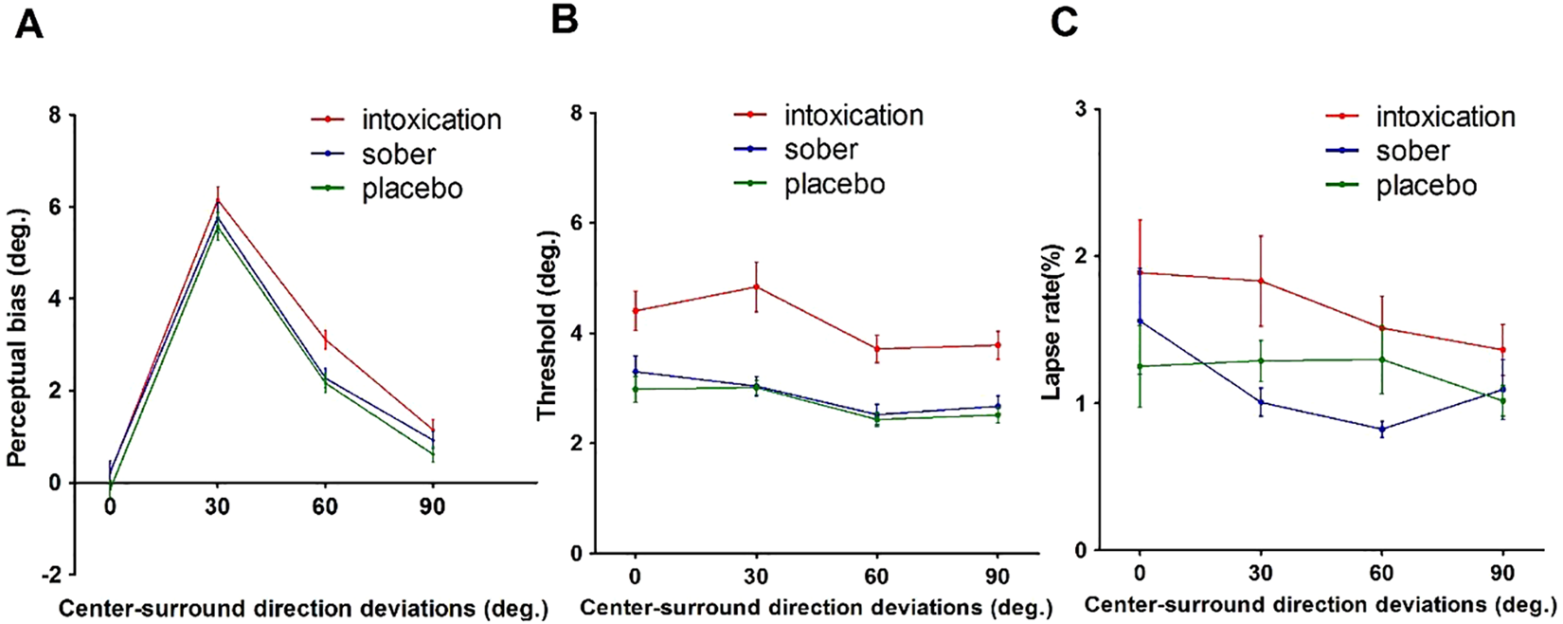
Motion repulsion results and lapse rates of sober, placebo and alcohol states. (**A**) Repulsion effects, indicated by the perceptual bias necessary to perceive the center as vertical, as a function of center-surround motion direction deviations (positive values indicate motion repulsion of the surround; the results for CW and CCW surrounds of same angular deviation were pooled). (**B**) Direction thresholds around perceived verticality. The mean values for the vertical discrimination thresholds as a function of the experimental condition. Error bars represent standard errors. (**C**) Lapse rates of various surround directions under sober, placebo and intoxication conditions.

We measured the magnitudes of motion repulsion as a function of direction differences between the reference and test directions under each condition for all participants (Fig. 3A). As expected, we successfully replicated the phenomenon of motion repulsion shown in previous studies^17,19–21^. The perceived direction was misjudged, in that the reported vertical was shifted relative to the true vertical direction (control condition, blue line). The amount of direction misperception for all three conditions was systematically modulated by the surround motion direction, consistent with previous reports. The misperception was most pronounced for center-surround motions with angular differences of 30° and 60°. Notably, the patterns of motion repulsion in the placebo and alcohol conditions were very similar to those in the sober condition, except that the motion repulsion (MR) magnitude in the alcohol intoxication condition exhibited a marked increase compared to the other conditions.

Repeated measures ANOVA with the surround direction factor (0°, ±30°, ±60° and ±90°) and conditional factor (sober, placebo and alcohol) revealed that there were significant main effects of center-surround direction differences on MR (F (3, 81) = 198.26, p < 0.001, ehat = 0.763) and the condition (F (2, 54) = 19.36, p < 0.001, ehat = 0.992), as well as a significant interaction between them (F (6, 162) = 2.76, p = 0.023, ehat = 0.789). This interaction effect was driven by a significant difference in the MR between the placebo and intoxication states with surround directions of 30° and 60°. Tests were then conducted under the placebo and intoxication conditions to identify significant differences at each test direction. We found a significant difference between both the surround direction (F (3, 81) = 181.97, p < 0.001, ehat = 0.808) and alcohol condition (F (1, 27) = 11.65, p = 0.002), as well as a significant interaction between them (F (3, 81) = 4.43, p = 0.011, ehat = 0.821). Compared to the placebo condition, the amplitudes measured in the intoxication condition were significantly higher, with a surround direction of ±60° (p < 0.01), but not ±0° (p = 0.74), ±30° (p = 0.37) or ±90° (p = 0.41).

### Discrimination performance

The psychometric functions provided estimates of both the perceived vertical and discrimination thresholds. These thresholds described the deviation of the motion direction from the perceived vertical at which subjects reported reliable deviations in 84% of trials. These deviations reflect the difficulty of discriminating two close directions of motion, with higher values indicating a worsened discrimination ability of the subjects. The average thresholds for each experimental condition are presented in Fig. 3B. The direction discrimination thresholds in the intoxicated state were larger than those in the sober and placebo conditions (F (2, 54 = 29.63, p < 0.001, ehat = 0.775), and the thresholds were modulated by the surround direction (F (3, 81) = 12.13, p < 0.001, ehat = 0.812). Importantly, there was no interaction between the condition and surround direction (F (6, 162) = 0.53, p = 0.697, ehat = 0.603), showing a similar trend as that of the threshold variation for different surround directions.

### Lapse rate and “high cognitive” effects

We first analyzed the lapse rates of the subjects. This variable indicated an overall attentional state of the subjects to the task and stimulus, which we suggest as an indicator of plausible attentional limit changes^22^ or deficits in the intoxicated condition. If “high cognitive” effects were present, it was suggested not only that subjects should have more lapses in the intoxication condition (less attention to stimulus presentation) but also that the lapses should be independent of the specific surround condition.

The average lapse rate for each experimental condition is presented in Fig. 3C. Repeated measures ANOVA of the surround direction factor (0°, ±30°, ±60° and ±90°) and conditional factor (sober, placebo and alcohol) was conducted. It revealed a significant main effect of the different conditions on the lapse rate (F (2, 54) = 5.55, p = 0.01, ehat = 0.848), while there was no difference between various surround directions (F (3, 81) = 2.13, p = 0.125, ehat = 0.706) or interaction effects (F (6, 162) = 0.78, p = 0.523, ehat = 0.575).

Another possibility is that the higher MR biases at 30° or 60° surround directions are due to possible confusion of the subjects, especially in the intoxicated state. When responding to the target, subjects may have instead responded to the surround direction of motion. If this was the case, then one should expect to see a correlation between the thresholds and biases for a given condition at a fixed surround of 30° or 60°. Pearson’s correlation was conducted between the perceptual bias and discrimination threshold. No correlation was found in the ±30° (Pearson r = 0.15, p = 0.24) or the ±60° (Pearson r = 0.15, p = 0.46) surround direction in the intoxication condition. These results demonstrate that changes in bias may not be related to “higher cognitive” effects.

## Discussion

In the current study, we investigated the effects of alcohol intoxication on motion repulsion before and after alcohol ingestion using human psychophysiological measures. We found that the magnitudes of motion repulsion in the intoxication state were significantly stronger compared to those of placebo controls. These results can be explained by altered inhibitory processing in motion-sensitive visual cortical areas during intoxication.

One of the main methodological concerns of studying behavioral differences before and after alcohol consumption is the generalized deficit in performance that is found in practically all tasks. This deficit is attributable to impairments in a number of cognitive and/or affective processes. Since alcohol is known to cause multiple cognitive and motor deficits, one might be concerned that the observed differences in the motion estimation task between the placebo and intoxication conditions are driven primarily by cognitive deficits or other general declines rather than by a sensory deficit. For example, participants in the intoxicated state may have a poorer understanding of the task or motor skills required to perform the task.

However, we believe that this explanation is not likely. In our data, the patterns of motion repulsion in the intoxication condition were very similar to those observed in the placebo condition. This indicates that the MR curves in the intoxicated state were modulated by the difference between the center and surround motion directions, similar to the placebo condition. Stated differently, acute moderate alcohol consumption affected the strength of motion repulsion while subjects reliably represented individual perceptual sensitivities. This replicated phenomenon of motion repulsion observed in the intoxication condition cannot be explained by deteriorated cognition or motor skills. Additionally, we found that motion repulsion differences were present in some directions, but disappeared when the actual angular separation was 0° or 30° (Fig. 3A). If generalized deficits play a role, then we should expect to observe a global influence, but this was not the case in our data.

Importantly, the overall changes in lapse rates indicate that our subjects had global changes in attention to the task. Our analysis revealed differences between lapse rates in the three conditions. While the intoxication condition showed an increase in lapse rate, this effect was global across surround orientations, indicating that our subjects had “high cognitive” effects unrelated to specific surround conditions. We also found no correlation between the bias and threshold at 30° and 60° surround directions, which also supports the idea that the motion repulsion changes observed in our results were due to low-levels of motion processing alterations that were caused by alcohol intake. In summary, we believe that the altered motion repulsion observed after alcohol ingestion reflects a specific deficit in visual motion processing, not a general cognitive effect.

Motion repulsion during direction judgments is often explained by inhibitory interactions between motion-tuned neurons^14–16^, particularly neurons in area MT/V5 tuned to visual unidirectional motion^16^. In our task, the motion direction repulsion was explained by neural inhibition. As a result, increased motion repulsion was likely to lead to higher neural inhibition in the system. Alcohol-related increases in motion repulsion could be explained by behavioral evidence, implying cortical alterations of inhibitory neural processing in motion-sensitive regions. Specifically, the neural inhibition among neurons responsible for the phenomenon of motion repulsion might have been altered under the alcohol intoxication condition. This interpretation is in line with the notion that ethyl alcohol, or ethanol, is linked to disrupted inhibitory functioning in the brain^23,24^. The current findings extend this concept by suggesting that alcohol intoxication is also involved in impaired inhibitory processing in visual motion cortical circuits. Prior investigations indicated that the predominant mechanism of central nervous system (CNS) depression involves selective alcohol interactions with ion channels that include an allosteric enhancement of inhibition that is mediated by gamma aminobutyric acid A (GABA-A) receptors, antagonism of excitation by N-methyl-D-aspartic acid (NMDA) glutamate receptors and, possibly, inhibition of central L-type Ca^2+^ channels^25^.

Recently, there was a report that showed that moderate acute alcohol intoxication had minimal effects on surround suppression measured with a sinusoidal grating-defined motion direction discrimination task^26^. Notably, this study used a between-subjects design, where subjects were assigned to an alcohol (n = 26) or control (n = 29) group. Because the final level of BAC is generally variable between individuals, this study may have missed strong within-subjects effects. Another explanation is that the dose administered was too small to produce detectable effects. The estimated BAC of the alcohol group (Fig. 3 in Read’s 2015 population results) was approximately 20 mg/100 ml, which is much lower than the BAC level (60 mg/100 ml) in our study. Thus, the previous study cannot exclude the possibility that larger doses would have produced changes in surround suppression.

In summary, our results suggest that moderate acute alcohol intoxication has significant effects on motion repulsion as measured by a RDK motion direction discrimination task. The results were consistent with findings that show suppression effects of ethanol on neural functioning by enhancement of inhibition mediated by gamma-aminobutyric acid A (GABA-A) receptors. Thus, we propose that the GABA-ergic system could account for the observed effects in our study.

A possible limitation of the study is the efficacy of placebo beverage administration. All participants had experience with drinking mixed alcoholic beverages. Given their familiarity with the timeline and subjective effects of alcohol metabolism, they might be able to discern the beverage content from a variety of sensations during the course of the entire session. Two participants reported that one of the two provided beverages tasted like null, though they were unaware of the alcohol content. Research in this area indicates that participants are typically “fooled” by placebo beverages and report that they consumed multiple alcoholic beverages following a placebo dosing beverage^27^. Moreover, the bolus dosing procedure used in the current study produced a profile (time-course, subjective, etc.) that was likely quite different from the typical drinking patterns of most (if not all) participants. Therefore, while the participants may have been able to indicate that the placebo beverage contained a lower amount of alcohol, they were likely unable to deduce that one of the two beverages did not actually contain alcohol.

## Methods and Materials

### Participants

The study consisted of 28 university students and staff (20 males, 20–30 years old, mean = 24.3 years) who did not report any somatic, neurological or psychiatric disease. Subjects completed a questionnaire before participating in the study to ensure that there was no problematic drinking or health conditions. All participants had experience with the dose of alcohol administered in the study, but they were neither binge drinkers nor regular drinkers. Those who reported consistent binge drinking at least once per week were not included in the study. Additionally, subjects were asked to abstain from both stimulants and sedatives, such as caffeine, nicotine, guanine, ethanol and benzodiazepines, for at least one day prior to the experiments. Participants were also asked to fast for one hour prior to alcohol administration. All subjects had normal or corrected-to-normal vision (mean decimal acuity was 1.3 ± 0.17) and were naive to the purpose of the experiment. This research was approved by the ethics committee of the University of Science and Technology of China and followed the guidelines of the Declaration of Helsinki. Written informed consent was obtained from each participant after explanation of the nature and possible consequences of the study, and they were paid hourly for their participation.

### Set-up

Stimuli were displayed on a 40.0 cm × 30.0 cm CRT monitor (Sony G520, Sony Corporation, Tokyo Japan; 85 Hz, resolution of 1600 × 1200 pixels) with self-programmed Matlab functions (Math works, Inc.) using the Psychophysics toolbox^28,29^. The experiment was conducted in a dimly illuminated room. To avoid local cues of the vertical/horizontal position, the screen was delimited by a 30.0 cm diameter circular window cut out of black cardboard. Luminance values were obtained with the help of the contrast box switcher^30^, which allowed the luminance range digitization to be extended above 10 bits. The eye-to-screen distance was maintained with a chin rest and fixed at 1.5 meters. Luminance values were obtained from a 256 RGB gray levels look-up table. Calibration was performed each day, and stimuli were viewed binocularly.

### Stimuli and procedure

Scenarios of the motion stimulus have been reported previously^31^ and consistently and clearly evoked significant motion repulsion effects in all participants^31^. Specifically, the stimulus was a center-surround structure filled with moving random-dot patterns (10 dots/square degree; coherence = 100%, each dot had speed of 8°/s, 0.1° diameter, and RGB value was 0; dot lifetime was infinite). The stimulus was presented on a mean background with a 45 cd/m^2^ luminance. The radius of the virtual circular aperture (target) was 1.5°, and the surround annulus virtual aperture had an inner/outer radius of 1.5/4.5° (context; Fig. 1). The stimulus in each trial was presented for 200 ms after a 200 ms fixation, and no feedback was provided. The surround direction of motion was defined with respect to the center target direction and was one of 7 predefined values, from −90° to +90° in increments of 30°. A surround of 0° had a motion direction equal to the central target motion direction. Positive and negative values corresponded to clockwise and counterclockwise directions from 0°, respectively (Fig. 1). The perceived direction (counterclockwise or clockwise relative to vertical upward) was reported by pressing left or right arrows on a keyboard. There were 560 trials (80 trials ×7 surround directions) in each task, and all conditions were pseudorandomly presented to each subject. The target motion direction was varied from trial to trial for measuring the perceived upward direction of motion under a surround motion direction. A weighted up-down adaptive procedure^32^ was used for psychometric curve measurement. For each surround direction, two staircases were assigned with up/down steps of 3/1 and 1/3 in steps of 1°, respectively. Each staircase contained 40 trials, with a starting direction of −15°/+15° positioned at the opposite side of the convergence point, allowing for rapid measurement within the transition region of the psychometric function.

Participants were instructed to fixate on a small black cross displayed at the center of the screen and were told that the stimulus would be presented there. Participants started each trial by pressing a button, and 200 ms after fixation cross disappearance, the whole stimulus (target + context) was presented for 200 ms. Subjects had to report whether the center target direction was tilted clockwise or counterclockwise from the internal vertical upward direction by pressing corresponding keys on the computer keyboard. No feedback was provided regarding response correctness.

### Experimental design

A within-subjects design was used to balance inter-individual differences in behavioral performances. Each subject was exposed three experimental conditions (no alcohol, placebo and moderate alcohol) on separate days. The dose of moderate alcohol was approximately 0.6 mg/ml. The no alcohol measure was conducted first. In both the placebo and alcohol conditions, subjects were told that they would receive alcohol. In the placebo condition, several drops of alcohol were floated on the top of each cup of juice to provide the taste and smell of alcohol. Thus, subjects in the placebo condition expected alcohol, but received only a negligible amount, while subjects in the alcohol condition both expected and received alcohol. The order of placebo and alcohol measures was counterbalanced to control for possible learning effects. During each experimental condition, subjects performed the same visual direction discrimination task. Additionally, the experiment was double-blind, in that neither the subjects nor the experimental data analyst were aware of the conditions.

Subjects were first weighed to calculate the appropriate dose of alcohol for each participant. Experiments and trials were initiated by subjects with a keyboard press. Subjects were requested to fixate on a small black square at the center of the screen. Before formal measurement, each subject received a short practice session (280 trials) for the direction discrimination task to rule out possible practice effects.

### Alcohol administration

Participants were served an individual amount of liquor (40% volume ethanol) mixed with equal proportions of orange juice to reach a BAC, when assuming an absorption deficit. The required amount of alcohol for each participant was calculated based on the following equation by Widmark (1932)^33^ and Watson *et al*. (1980)^34^:

1 $${c}=\frac{0.8\times {A}}{1.055\times {TBW}}$$

where *c* denotes the maximum possible BAC milliliter and was set to 1.5. Since the equation does not take the absorption deficit into account, the final BAC value for each subject was determined by an Alcotest measurement. *TBW* is the total body water in liters and was estimated using different equations for men and women to account for differences in body fat:

2 $$TBW\,{\rm{women}}=-2.097+(0.1069\times {h})+(0.2466\times {w})$$

3 $$TBW\,{\rm{men}}=2.447-(0.09516\times a)+(\mathrm{0.1.74}\times h)+(0.3362\,\times w)$$

where *h* is the body height in cm, *w* is the body weight in kg, and *a* is the age in years.

*A* is the amount of alcohol in grams that must be consumed. To determine an individual value for *A*, the equation was transformed to:

4 $$A=\frac{1.055\times TBW\times c}{0.8}$$

Finally, the amount of alcoholic beverage in ml (*V*) was calculated using the equation:

5 $$V=\frac{A}{(vol\div100)\times 0.8}$$

where *vol* is the % volume of the alcoholic beverage and was set to 40. *V* is the final amount (ml) of alcohol that subjects consumed in the experiment. The same volume of juice was mixed with alcohol for administration. In the present study, the mean amount of liquor intake was 154 ml (±28 ml). Irrespective of the individual amount, ingestion of ethanol had to be completed within 15 minutes.

Before the experiment began, the BAC was measured with an Alcotest 6510 breathalyzer (Drägerwerk, Lübeck, Germany) to ensure a BAC of 0 mg/ml. The BAC was measured continually (except during task performance) starting ten minutes after consumption of all alcoholic beverages, including in the placebo condition. The experimental procedure was initiated after 30 additional minutes, so that participants performed under peak BAC conditions. The BAC was measured for both the alcohol and placebo conditions.

### Data Analysis

We performed data analysis using custom-made MATLAB scripts. The magnitude of motion repulsion at each test direction was determined as the angular difference between the perceived and physical directions^19,20^. The raw data of each surround motion and condition were fitted with a logistic function that consisted of computing the proportion of clockwise responses as *p*_*i*_ = *y*_*i*_/*n*_*i*_, where *n*_*i*_ is the number of occurrences, *x*_*i*_ is the target motion direction, and *y*_*i*_ is the number of clockwise responses. The psychometric function was logistically defined as:

6 $${\rm{p}}(x)=l+\frac{1-2l}{1+exp(-\frac{log(21/4)}{\sigma }(x-\mu ))}$$

where *l* is the lapsing rate of subjects, *μ* is the midpoint, i.e. the motion direction perceived as vertical upward, and *σ* is the threshold needed to perceive a deviation from the reference point (>84% correct responses). The function was adjusted to the data by using Bayesian fitting^35^. Prior parameters were: *l*-beta probability distribution with parameters Beta (1.2, 15); *σ*-gamma probability distribution with parameters Gamma (2.5, 2.5); and μ had a uniform prior. The midpoint of a given block of measures were then adjusted to a mean of zero by subtracting the average. The midpoint (*μ*), threshold (*σ*) and lapse (*l*) were extracted using the above methods for each subject, surround direction, and condition.

We conducted repeated measures ANOVA on the magnitudes of repulsion, with the test direction (0°, ±30°, ±60° and ±90°) and different conditions (sober, placebo and alcohol) as the within-subject factors as well as with the Geisser-Greenhouse adjusted statistics (epsilon is reported as ehat). The sober (no alcohol) state was intended to replicate the phenomenon of motion repulsion, allowing us to compare it with previous studies to validate our methods. The main group difference that we wanted to explore was between the placebo and alcohol conditions. For the current report, we used the second MR measure obtained around the peak intoxication level, as demonstrated by the BAC analysis shown at the beginning of the Results section. We also performed Bonferroni post-hoc multiple comparisons for the repulsions at each test direction.
