# Author Correction: Moderate acute alcohol intoxication increases visual motion repulsion

**DOI:** 10.1038/s41598-019-44354-5

**Published:** 2019-05-23

**Authors:** Zhengchun Wang, Huan Wang, Tzvetomir Tzvetanov, Yifeng Zhou

**Affiliations:** 1 Department of Physiology and Pharmacology, Medical School of Ningbo University, Ningbo, Zhejiang 315211 P.R. China; 2 Hefei National Laboratory for Physical Sciences at Microscale and School of Life Science, University of Science and Technology of China, Hefei, Anhui 230027 P.R. China; 3 School of Computer and Information, Hefei University of Technology, Hefei, Anhui 230009 P.R. China; 4 State Key Laboratory of Brain and Cognitive Science, Institute of Biophysics, Chinese Academy of Science, Beijing, 100101 P.R. China

Correction to: *Scientific Reports* 10.1038/s41598-018-19932-8, published online 25 January 2018

This Article contains errors.

In reprocessing the data for further analyses, the Authors found that the data presented in the Article was not processed as stated in the Methods. The motion repulsion data of alcohol condition used in the article were selected based on the strength of motion repulsion effects. We chose the alcohol measure which had the visually strongest repulsion effects as the alcohol condition data. As a result, some of the Results differ to what is presented in the Article, although we find that the overall qualitative result and the conclusions of the study are unaffected.

The Article is being corrected as follows.

In the Methods and Materials section, under the subheading “Participants”:

“The study consisted of 28 university students and staff (20 males, 20–30 years old, mean = 24.3 years) who did not report any somatic, neurological or psychiatric disease.”

should read:

**“**The study consisted of 33 university students and staff (24 males, 20–30 years old, mean = 23.94 years).”

At the end of the Methods and Materials section, under the subheading “Experimental Design”, the following sentence should be included:

“Additionally to motion repulsion measures, interleaved with it were performed measures of center-surround Orientation repulsion. They are not reported here but analyzed in a separate work.”

At the beginning of the Methods and Materials section, under the subheading “Data Analysis”, the following paragraph should be included:

“From the 33 participants, 5 subjects did not have a full data set (at least one alcohol measure, or control, or placebo measures) for one of the following reasons: did not want to drink such amount of alcohol and decision was taken to abort measures with these persons (3), missed Control measures due to availability of the persons (1), and left to another city for work (1). From the 28 subjects with valid data sets, 26 had at least two alcohol measures and 21 subjects at least 3 alcohol measures. The number of alcohol measures depended on individual subject’s well-being during those measures.”

At the end of the Methods and Materials section, under the subheading “Data Analysis”, the following sentence should be included:

“Repeated measures ANOVA was applied on the extracted threshold and log10-transformed lapse rates.”

Results shown in Figure 3 of this Article are corrected following the re-analysis. The corrected Figure 3 is shown as Figure 1.

**Figure 1:**
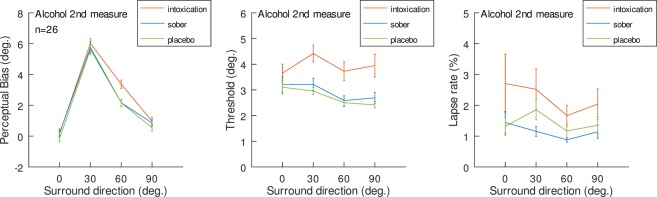
Motion repulsion results and lapse rates of sober, placebo and alcohol states. (**A**) Repulsion effects, indicated by the perceptual bias necessary to perceive the center as vertical, as a function of center-surround motion direction deviations (positive values indicate motion repulsion of the surround; the results for CW and CCW surrounds of same angular deviation were pooled). (**B**) Direction thresholds around perceived verticality. The mean values for the vertical discrimination thresholds as a function of the experimental condition. Error bars represent standard errors. (**C**) Lapse rates of various surround directions under sober, placebo and intoxication conditions.

In the Results section, under the subheading “Increased motion repulsion after alcohol administration”, the following paragraph:

“Repeated measures ANOVA with the surround direction factor (0°, ±30°, ±60° and ±90°) and conditional factor (sober, placebo and alcohol) revealed that there were significant main effects of center-surround direction differences on MR (F (3, 81) = 198.26, p < 0.001, ehat = 0.763) and the condition (F (2, 54) = 19.36, p < 0.001, ehat = 0.992), as well as a significant interaction between them (F (6, 162) = 2.76, p = 0.023, ehat = 0.789). This interaction effect was driven by a significant difference in the MR between the placebo and intoxication states with surround directions of 30° and 60°. Tests were then conducted under the placebo and intoxication conditions to identify significant differences at each test direction. We found a significant difference between both the surround direction (F (3, 81) = 181.97, p < 0.001, ehat = 0.808) and alcohol condition (F (1, 27) = 11.65, p = 0.002), as well as a significant interaction between them (F (3, 81) = 4.43, p = 0.011, ehat = 0.821). Compared to the placebo condition, the amplitudes measured in the intoxication condition were significantly higher, with a surround direction of ±60° (p < 0.01), but not ±0° (p = 0.74), ±30° (p = 0.37) or ±90° (p = 0.41).”

should read:

“Repeated measures ANOVA with the surround direction factor (0°, ±30°, ±60° and ±90°) and conditional factor (sober, placebo and alcohol) revealed that there were significant main effects of center-surround direction differences on MR (F(3,75) = 169.7, p < 0.001, ehat = 0.764) and the condition (F(2,50) = 12.81, p < 0.001, ehat = 0.991), as well as a significant interaction between them (F(6,150) = 4.58, p = 0.0007, ehat = 0.832). This interaction effect was driven by a significant MR increase in intoxication state at surround direction of 60°. Tests were then conducted under the placebo and intoxication conditions to identify significant differences at each test direction. Compared to the placebo condition, the amplitudes measured in the intoxication condition were significantly higher at surround direction of 60° (t(25) = −5.27, p < 0.001) and 90° (t(25) = −2.70, p = 0.0121), but not at 0° (t(25) = −2.33, p = 0.028) and 30° (t(25) = −1.43, p = 0.16) (Bonferroni correction of significance level to 0.05/4 = 0.0125).”

In the Results section, under the subheading “Discrimination performance”, the following paragraph:

“The direction discrimination thresholds in the intoxicated state were larger than those in the sober and placebo conditions (F (2, 54 = 29.63, p < 0.001, ehat = 0.775), and the thresholds were modulated by the surround direction (F (3, 81) = 12.13, p < 0.001, ehat = 0.812). Importantly, there was no interaction between the condition and surround direction (F (6, 162) = 0.53, p = 0.697, ehat = 0.603), showing a similar trend as that of the threshold variation for different surround directions.”

should read:

**“**The direction discrimination thresholds in the intoxicated state were larger than those in the sober and placebo conditions (F(2,50) = 12.21, p = 0.0005, ehat = 0.684), and the thresholds were modulated by the surround direction (F(3,75) = 8.44, p = 0.0002, ehat = 0.867). There was a trend of interaction between the condition and surround direction (F(6,150) = 2.50, p = 0.056, ehat = 0.581), which was driven by differences in thresholds at 30°, 60°, 90° but not at zero degrees surrounds (placebo vs. Alcohol: at 0° − t(25) = −1,65, p = 0.11; at 30° − t(25) = −4.76, p < 0.0001; at 60° − t(25) = −3.73, p = 0.001; at 90° − t(25) = −3.60, p = 0.0014).”

In the Results section, under the subheading “Lapse rate and high cognitive effects”, the following sentence:

“It revealed a significant main effect of the different conditions on the lapse rate (F (2, 54) = 5.55, p = 0.01, ehat = 0.848), while there was no difference between various surround directions (F (3, 81) = 2.13, p = 0.125, ehat = 0.706) or interaction effects (F (6, 162) = 0.78, p = 0.523, ehat = 0.575).”

should read:

“It revealed a significant main effect of the different conditions on the lapse rate (F(2,50) = 8.04, p = 0.0037, ehat = 0.695), a main effect of various surround directions (F(3,75) = 4.31, p = 0.009, ehat = 0.917), but no interaction effects (F(6,150) = 0.40, p = 0.84, ehat = 0.791).”

and in the same section:

“If this was the case, then one should expect to see a correlation between the thresholds and biases for a given condition at a fixed surround of 30° or 60°. Pearson’s correlation was conducted between the perceptual bias and discrimination threshold. No correlation was found in the ± 30° (Pearson r = 0.15, p = 0.24) or the ± 60° (Pearson r = 0.15, p = 0.46) surround direction in the intoxication condition. These results demonstrate that changes in bias may not be related to “higher cognitive” effects.”

should read:

“We note that if this was the case, the main effect of such a task change would be to shift the psychometric functions (midpoints) in opposite directions from the repulsion effects. This would in fact decrease the repulsion effect in intoxicated condition when compared to control and placebo conditions, an effect that our data did not show. On the other hand, intoxicated condition might simultaneously influence bias and thresholds due to either low-sensory effects or high-cognitive effects, thus showing correlated changes in both measures across subjects. We computed the Pearson’s correlations between the thresholds and biases for each condition and surround. Among the 12 correlations (3 conditions x 4 surrounds), two showed significant correlations (Alcohol at 60°: r = 0.55, p = 0.0039; placebo at 90°: r = 0.55, p = 0.0032) after adjustment for multiple tests (Bonferroni adjustment to 0.05/12 = 0.0042) (all others p > 0.01). These results suggest that changes in bias and threshold may not be related to “higher cognitive” effects in intoxicated condition only.”

Additionally, we re-analyzed the data for all orders of Alcohol measure and we found that the main claims of the study are still valid, irrespective of which Alcohol measure is considered - 1^st^, 2^nd^ or 3^rd^. Below, we provide results of the analysis as applied in the main article for measures number 1 and number 3 (measure number 2 is presented in Figure 1 of this Correction).

From the 28 persons with at least one Alcohol measure, the Bayesian fitting procedure of psychometric function parameters extraction gave in one measure A1 (Alcohol measure 1) two lapse rates above 0.25. Inspecting the staircases of the subject showed that the person responded to the surround direction instead of the center target for about half of the trials. No other such case was present among the remaining 27 subjects’ staircases. Thus, for alcohol measure 1 there were 27 data sets, for alcohol measure 2 there were 26 data sets and alcohol measure 3 there were 21 data sets. Figure 2 presents the results of each analysis.

**Figure 2:**
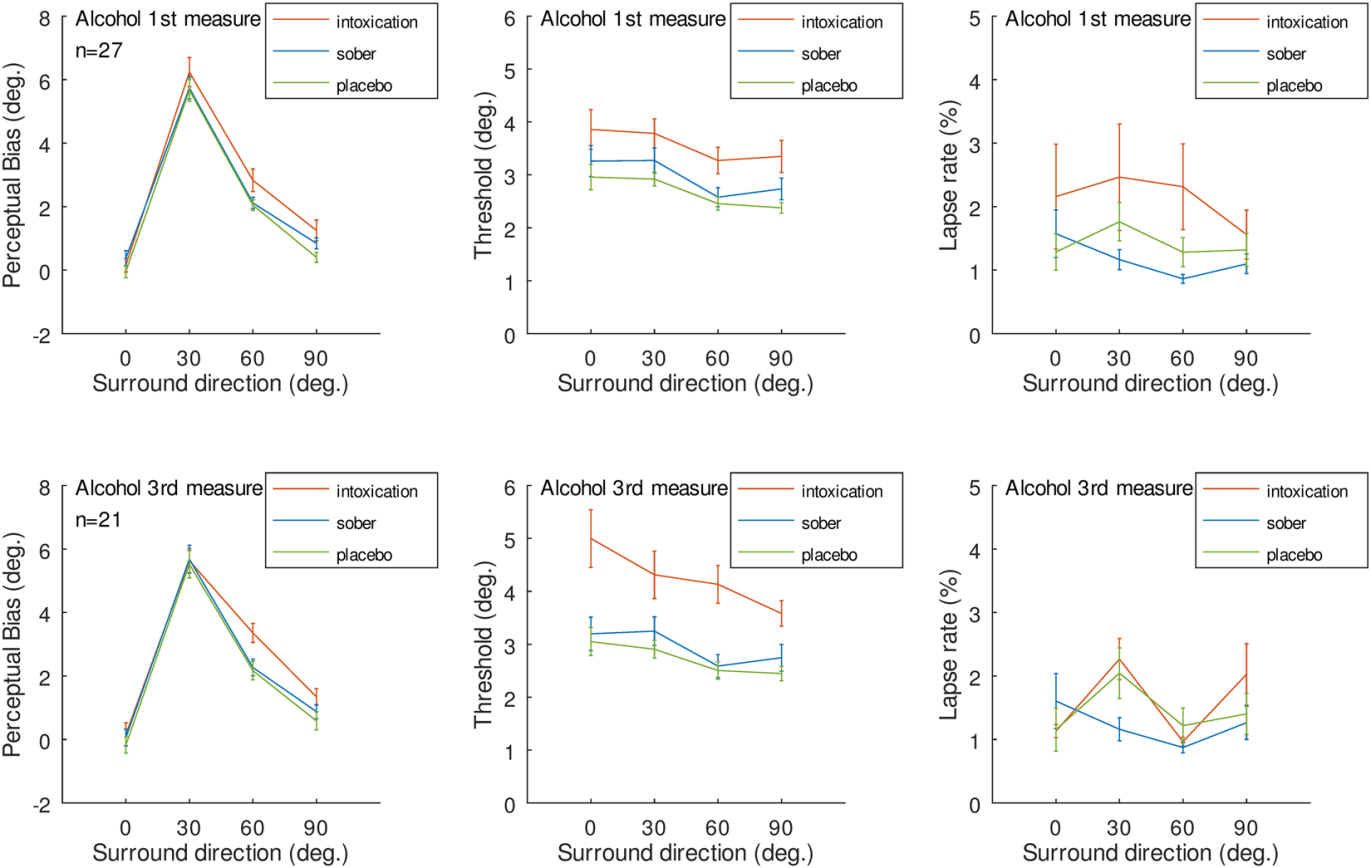
Results for bias, threshold, and lapse rates for 1^st^ and 3^rd^ measures of alcohol. Same format as Figure 3 in main text.

The ANOVA analyses are presented in Table 1. We note that (1) for Bias measures Condition always showed significant effect with Alcohol measure giving stronger repulsion, (2) Thresholds were always higher in Alcohol measure across all three measures, (3) the lapse rates were higher in alcohol conditions for all three measures and did not interact with surround direction.

**Table 1:** ANOVA table results for each Alcohol measure.

	Measure	Condition	df	F	Sig.	ehat
First Alcohol measure (n = 27)	Bias	C	2, 52	6.16	0.009	0.762
		S	3, 78	215.84	<0.001	0.697
		C*S	6, 156	1.51	0.23	0.380
	Threshold	C	2, 52	8.01	0.003	0.723
		S	3, 78	10.5	0.002	0.655
		C*S	6, 156	0.23	0.94	0.770
	Lapse	C	2, 52	5.07	0.018	0.741
		S	3, 78	1.98	0.14	0.811
		C*S	6, 156	1.02	0.41	0.792
Second Alcohol Measure (n = 26)	Bias	C	2, 50	12.81	<0.0001	0.991
		S	3, 75	169.67	<0.0001	0.764
		C*S	6, 150	4.58	0.0007	0.832
	Threshold	C	2, 50	12.21	0.0005	0.684
		S	3, 75	8.44	0.0002	0.867
		C*S	6, 150	2.50	0.0558	0.581
	Lapse	C	2, 50	8.04	0.004	0.695
		S	3, 75	4.31	0.009	0.917
		C*S	6, 150	0.40	0.84	0.791
Third Alcohol Measure (n = 21)	Bias	C	2, 40	10.93	0.0002	0.997
		S	3, 60	97.1	<0.0001	0.717
		C*S	6, 120	2.46	0.060	0.593
	Threshold	C	2, 40	16.77	0.0001	0.716
		S	3, 60	11.59	0.0001	0.635
		C*S	6, 120	1.97	0.108	0.656
	Lapse	C	2, 40	6.14	0.006	0.940
		S	3, 60	8.67	0.0001	0.923
		C*S	6, 120	2.09	0.088	0.683

The Authors apologize for the errors, and any inconvenience caused.

